# Innovative treatment formats, technologies, and clinician trainings that improve access to behavioral pain treatment for youth and adults

**DOI:** 10.3389/fpain.2023.1223172

**Published:** 2023-07-20

**Authors:** Beth D. Darnall, Karlyn A. Edwards, Rena E. Courtney, Maisa S. Ziadni, Laura E. Simons, Lauren E. Harrison

**Affiliations:** ^1^ Stanford Pain Relief Innovations Lab, Department of Anesthesiology, Perioperative and Pain Medicine, Stanford University School of Medicine, Palo Alto, CA, United States; ^2^ Salem VA Health Care System, PREVAIL Center for Chronic Pain, Salem, VA, United States; ^3^ Virginia Tech Carilion School of Medicine, Department of Psychiatry and Behavioral Medicine, Roanoke, VA, United States; ^4^ Systems Neuroscience and Pain Lab, Department of Anesthesiology, Perioperative and Pain Medicine, Stanford University School of Medicine, Palo Alto, CA, United States; ^5^ Biobehavioral Pediatric Pain Lab, Department of Anesthesiology, Perioperative and Pain Medicine, Stanford University School of Medicine, Palo Alto, CA, United States

**Keywords:** chronic pain, acute pain, psychological, treatment, behavioral, digital, education, pediatric

## Abstract

Chronic pain is prevalent across the life span and associated with significant individual and societal costs. Behavioral interventions are recommended as the gold-standard, evidence-based interventions for chronic pain, but barriers, such as lack of pain-trained clinicians, poor insurance coverage, and high treatment burden, limit patients’ ability to access evidenced-based pain education and treatment resources. Recent advances in technology offer new opportunities to leverage innovative digital formats to overcome these barriers and dramatically increase access to high-quality, evidenced-based pain treatments for youth and adults. This scoping review highlights new advances. First, we describe system-level barriers to the broad dissemination of behavioral pain treatment. Next, we review several promising new pediatric and adult pain education and treatment technology innovations to improve access and scalability of evidence-based behavioral pain treatments. Current challenges and future research and clinical recommendations are offered.

## 1. Introduction

Chronic pain is a global health crisis, affecting more than 20% of people worldwide (1, 2). The personal and economic burden of chronic pain is striking, with known impact on the individual and social contexts (e.g., parents, partners, employers) and societal costs exceeding 650 billion USD annually for pediatric and adult pain treatment costs and lost productivity (1, 2). Given the biopsychosocial nature and impacts of chronic pain, a multidisciplinary, person-centered treatment approach yields the best pain treatment outcomes at the lowest cost (3–8). Conceptualization of chronic pain through a biomedical lens, as well as the absence of behavioral approaches, can lead to overmedicalization and reliance on costly procedures, surgery, and pharmacology, which are options that carry substantial health risks and may be ineffective for relieving pain. In addition to being recommended as a best practice for pain treatment (7–12), behavioral approaches are notable because they are non-pharmacologic and non-invasive and have a very low-risk profile. While a number of effective behavioral pain interventions exist for youth and adults with chronic pain (13, 14), access is limited by multiple factors, including a shortage of treatment services outside of urban areas, significant treatment-related costs, long provider waitlists, and a lack of clinicians trained in behavioral pain management (12, 15, 16). Treatment burden is another formidable barrier. Most behavioral pain treatments involve multiple sessions, resulting in ∼16–20 h of total treatment time (e.g., cognitive behavioral therapy for chronic pain, acceptance and commitment therapy, and mindfulness-based stress reduction). Thus, there is a need for innovative, efficient, and scalable behavioral intervention formats for treating pain.

The multiple critical barriers to behavioral pain care have been recognized at the federal level, with the US Health and Human Services (HHS) National Pain Strategy (17), the Federal Pain Research Strategy (2), the Interagency Best Practices Pain Management Task Force (15), and the Centers for Disease Control and Prevention calling for better integration of behavioral pain treatments into national pain care pathways. To address the care gap, HHS has called for more robust and widespread training for behavioral clinicians in chronic pain and related sequelae. The Federal Pain Research Strategy and HHS also called for better study of behavioral pain treatments that leverage technological solutions to potentially scale pain treatments broadly and support improved patient access to care (2, 15).

To better understand the problem, in this scoping review, we first review data describing system-level barriers to broad dissemination of behavioral chronic pain treatment. Next, we review several promising new pediatric and adult pain education and treatment innovations that leverage technology to improve and scale access to evidence-based behavioral pain treatments that complement a patient's treatment plan or serve as a standalone intervention. Finally, this article points to current and future challenges and offers recommendations for clinical targets and future research.

## 2. System-level barriers to dissemination of behavioral chronic pain treatment

An important system-level barrier involves a biomedical culture of pain management in many Western countries. This culture permeates medical education and clinical care and can impede patient access to the needed behavioral and psychological services due to a lack of understanding about the importance of the biopsychosocial treatment model and downstream consequences (e.g., patients are not explained with the role of psychology and the importance of behavioral treatments in their pain care plan and a lack of in-house referral options and/or professional connections in the community).

In 2016, the American Academy of Pain Medicine (AAPM) Pain Psychology Task Force published the results of a multi-stakeholder US survey (*N* = 1,991) that assessed barriers to behavioral chronic pain treatment (i.e., pain psychology), including system-level barriers and needs related to pain education and training of clinicians (16). Survey responses were received from a total of 1,991 respondents, including 1,086 patients, 843 clinicians (psychologists/therapists, *n* = 323; pain physicians, *n* = 203; primary care physicians and physician assistants, *n* = 221; nurse practitioners, *n* = 96), and 62 graduate and post-graduate psychology training directors. For patients, costs and insurance coverage were cited as barriers to pain psychology services. Moreover, over a third of the sample cited insufficient access to pain-trained therapists, as well as not knowing how to locate skilled providers. Supporting these findings, a recent examination of US insurance claims data of adults with chronic low back pain (*N* = 55,945) found only 4% utilized psychological therapy due to high out-of-pocket costs and poor insurance coverage (18). One small study suggested that even when pediatric patients receive specialty evaluation in a multidisciplinary pain clinic, less than half ultimately engaged in the recommended behavioral treatment, citing “not interested” as the primary reason for their lack of engagement (19). Similarly, roughly one-quarter of community-based adults with chronic pain reported being disinterested in behavioral pain treatment because they understood their pain was “not psychological” and/or behavioral pain treatment “would not help” (16). Disinterest in behavioral or psychological approaches has been described by others (20, 21) and may be partially driven by stigma (8).

Stigma is a key barrier to treatment engagement and is a fundamental cause of health inequities (22). Pain-related stigma is commonly experienced among adolescents and adults when seeking medical care, such as having their pain dismissed, disbelieved, or perceived to be exaggerating their pain. These experiences are known to worsen pain, mental health, social isolation, and treatment engagement over time (23, 24). Marginalized communities, such as women, gender and sexual minorities, and racialized patients, experience greater stigma and discrimination when seeking medical care further worsening health outcomes (25). Compounding these experiences, many people describe resistance to engaging in behavioral treatments for fear of confirming harmful messages that their pain is not real or “all in their head” (26, 27). Patients also report mixed reactions to psychological explanations of pain (e.g., stress, emotions, and thoughts can worsen pain), with some noting that this messaging conveys a sense of control, while others felt stigma and shame about their mental health (27). Given that many patients experience stigma and invalidation, clinicians trained in-patient-centered communication strategies and behavioral pain interventions are vital to improving treatment engagement and patient wellbeing.

While system-level barriers (e.g., high cost, poor insurance coverage, stigma) are key drivers, poor access to pain psychology services is also a result of the lack of mental health clinicians who are trained and skilled in delivering behavioral treatment for chronic pain. Indeed, the Pain Psychology Task Force survey found that among graduate and post-graduate psychology training directors, 36% (*n* = 21) reported that their programs offered no training on health or pain management. Of the programs that included pain in their graduate curriculum, 32% (*n* = 8) reported offering 1–4 h of pain content, 40% (*n* = 10) reported 5–10 h of pain content, and 28% (*n* = 7) reported ≥11 h of pain coursework and instruction. Insufficient pain training is also evident in the survey results of clinical psychologists and mental health therapists wherein 88% (*n* = 311) reported poor confidence and/or competency to treat pain due to a lack of graduate and professional pain training. Historically, physician training has also lacked curricula on the assessment and treatment of chronic pain and its psychosocial sequelae (28–30).

The Pain Psychology Task Force survey revealed broad patient and clinician stakeholder support for increased training in pain psychology. Most patients surveyed stated they would (66%) or might be (21%) in favor of an initiative to train more therapists to provide quality pain psychology services. The vast majority of medical clinician respondents (84%–95%) reported a need for pain-trained behavioral health clinicians. Moreover, they supported a national effort to accomplish this training goal and stated that pain psychology services would benefit their patients with chronic pain. Finally, all graduate psychology training directors surveyed (*n* = 55) reported being interested in integrating a brief, high-quality, packaged pain psychology training into their doctoral programs if offered at no cost.

In summary, within the context of pain and in the USA specifically, patient access to psychology services is poor. High cost, poor insurance coverage, stigma, and a lack of trained mental health clinicians to deliver behavioral pain treatment are system-level barriers to address. Barriers to care will differ based on country; for instance, within Canada and the UK, patients may experience general health service wait times of 1–3 years. While evidence-based treatments should be promoted whenever available and feasible, the following section describes several innovative approaches that are helping shore clinician training gaps and provide the public and patients with new and convenient ways to receive pain education and care. Such novel educational and treatment options do not obviate the need for evidence-based pain care; rather, they can be useful and necessary in cases where other treatments are inaccessible or unwanted, to supplement existing therapies, and to provide general pain education to various stakeholder groups. These new approaches include digitally delivered pain education and support, scalable clinician trainings, innovative efficiencies in integrative care, and direct access to evidence-based behavioral pain treatments.

## 3. Innovations supporting accessible chronic pain education, clinician training, and behavioral pain treatment

### 3.1. Digitally delivered pain education and resources

Digital innovations, such as videos, podcasts, and web-based applications (apps), have increased public access to pain education and support. Table 1 provides a non-exhaustive listing of key public and patient pain education resources.

**Table 1:** Innovative digital strategies to enhance behavioral pain education, and scalable clinician resources and certifications.

Resources/trainings	Public resource	Patient/family resource	Clinician resource
Brief pain science videos [e.g., Tame the Beast (31–33), TED-Ed Mysterious Science of Pain]	X	X	
Podcasts [e.g., *One Thing* (34)]	X	X	
Free and commercial Apps [e.g., *Manage My Pain* (35); *Curable* (36)]	X	X	
VA ECHO Program (37)			X
*Empowered Relief* 2-day clinician certification workshop (38)			X
*Comfort Ability* site certification (39)			X

A recent study systemically reviewed YouTube video resources focused on pain neuroscience education and identified 17 videos that addressed as least one target concept of pain education very well (31). One video, “Tame the Beast: It's Time to Rethink Persistent Pain,” addressed all target concepts of pain education (32). This short (5-min) animated video explains the difference between acute and chronic pain, emphasizes the conditioning that occurs in the brain in the context of chronic pain, and provides a rationale for behavioral pain treatment. Additional video resources showing merit were posted by educational organizations like TED-Ed who partnered with academic and healthcare professionals to create highly engaging and accessible content. Video resources posted on this platform are particularly promising for delivering pain education that can be freely accessed on YouTube.com by clinicians, patients, and the public.

Other novel efforts leverage technology to support public dissemination of pain education. *One Thing* was created in 2020 by a team of pain scientists and is a platform where well-established pain researchers and clinicians can discuss “one thing” they want others to know about chronic pain, including the latest research and tips/tricks for engagement in pain treatment. *One Thing* enduring content is available in video and podcast formats (34). Other educational podcasts include *Comfort Ability* (40), an audio podcast that includes tips and skills for managing pediatric pain, and conversations with teens about their experiences with chronic pain and treatment.

The Curable app is a commercial monthly subscription product that provides audio-based pain education and pain management content, lectures, and tools (36).

The Manage My Pain app is a free customizable digital tracking and pain education platform that helps patients and doctors better measure and monitor pain so it can be better managed (35).

### 3.2. Scalable clinician trainings

More than a decade ago the Veterans Health Administration (VHA) developed the Specialty Care Access Network- ECHO program. ECHO offers clinicians across the US virtual standalone continuing education telementoring sessions on pain-related topics and integrative case consultation with content experts (37). ECHO helps overcome primary care providers’ geographic barriers to specialty care. ECHO supports the increased use of non-opioid medications and rehabilitative services for chronic pain.

Beyond case consultations, many clinicians—both pain-trained and not—seek to efficiently address their clientele's behavioral pain treatment needs with efficient and standardized treatments. The *Empowered Relief* Clinician Certification Workshops are 2-day (11 h) interprofessional workshops that certify licensed clinicians of any discipline to deliver a one-session skill-based pain relief intervention (“Empowered Relief”) (38). The *Empowered Relief* intervention is didactic and group-based, with highly standardized content (PowerPoint deck and an instruction manual with full scripts) and intervention fidelity supports. Moreover, it is suitable for clinicians with minimal prior pain training. Certified clinicians may integrate this packaged and brief intervention into healthcare settings of all types. The clinician certification workshops are online and available internationally.

Within the Veterans Healthcare Administration (VHA), thousands of healthcare students receive education and training in best practices. The VHA also offers national trainings for clinicians and students to implement treatments such as eight-session cognitive behavioral therapy for chronic pain (CBT-CP) and briefer versions designed to be integrated into primary care clinics (brief CBT-CP) (41). Technology has further allowed the VHA to educate their providers in pain neuroscience, as well as consult with experts in the field to provide better care for patients.

While current scalable clinician trainings in Table 1 are promising, they are notably narrow in scope and number. Varied efficient and practical clinician trainings are needed to shore clinical competencies and expand patient access to evidence-based pain care.

### 3.3. Direct access to evidence-based behavioral pain treatments

Technological advances that directly increase patient access to pain care include AI-assisted cognitive behavioral therapy (CBT) for chronic pain, a one-session pain relief skill intervention (*Empowered Relief*) for acute and chronic pain, on-demand virtual reality (VR) device treatment for acute and chronic pain across the life span, and app-based interventions that teach cognitive and behavioral pain management skills (WebMAP Mobile; iGET Living) and opioid misuse in adults (*Empowered Relief* On-Demand).

The largest and most varied advances have occurred in behavioral pain interventions, some of which involve telehealth applications. A recent review of qualitative studies of enablers and barriers to telehealth interventions for people with chronic pain found that interventions with well-designed interactive platforms, flexibility to fit patients’ routine, and the broad availability of material favor better engagement (42). Moreover, encouragement of self-efficacy is linked to successful telehealth-delivered self-management programs.

As outlined in Table 2, advances include efficient clinician-delivered youth–family interventions with and without telehealth supports, a range of clinician-delivered one-session interventions, and various digital “on-demand” treatments that do not require a therapist.

**Table 2:** Innovative clinician-delivered treatments/interventions and interventions accessed through the medical setting (blue text for youth interventions).

	Public resource	Patient/family resource	Clinician-delivered intervention	Evidence-based	Format
*Comfort Ability* 1-day youth patient and family workshop (39) with corresponding website resource tools		X	X	X (43)	In-person or online
iGetLiving (44)		X	x		Digital
iCanCope (45)	X	X	x	X (46)	Digital
WebMAP (8-session online CBT for youth and families)	X	X	X	X (47–49)	Digital
SurgeryPal^™^ (online multi-session for youth undergoing surgery and family) (50)		X	X		Digital
Multi-session brief CBT for chronic pain (CBT-CP)			X	X (51)	In-person
One-session *Empowered Relief* intervention for chronic pain	X	X	X	X (52–54)	In-person or online
Digital On-Demand *Empowered Relief* for Surgery		X	X	X (55, 56)	Digital
One-session Emotional Awareness and Expression Training for chronic pain		X	X	X (57, 58)	In-person or online
One-session Mindfulness-Oriented Recovery Enhancement for chronic pain		X	X	Abstract (59)	In-person or online
Pain psychology and pain neuroscience self-evaluation intervention	X	X		X (60)	Digital
AI-assisted CBT		X	X	X (61)	Phone and digital
PainTrainer (on-demand self-paced CBT skills) (62)		X		X (63)	Digital
Virtual Reality Treatment for Chronic Pain^a^		X		X (64–68)	Digital device
Text support for opioid tapering (69)		X			Digital
Cancer pain management app (70, 71)		X			Digital

^a^ Clinician-prescribed; home-based self-administered treatment device.

### 3.4. Clinician-delivered or clinician-assisted interventions that expand treatment access

*Brief cognitive behavioral therapy for chronic pain (brief CBT-CP)* typically involves 8–10 weekly treatment sessions. CBT targets increased patient engagement in pleasant activities, decreasing maladaptive cognitions such as catastrophic appraisals, and reducing arousal with relaxation training. Data from multiple chronic pain studies suggest that CBT decreases pain intensity and pain catastrophizing and increases mood, mindfulness, physical function, self-efficacy, and pain acceptance (14, 52, 72–74). It was designed to be integrated into primary care clinics and increase access to evidence-based pain care. Within US Veteran healthcare, full protocol CBT-CP involves 3 months of weekly hour-long sessions (12 h total); in contrast, the brief CBT-CP is a targeted, manualized treatment that consists of six or fewer 30-min sessions (3 h total) and is delivered either in-person in primary care clinics or via telehealth. Brief CBT-CP improves pain function, as is associated with high patient satisfaction and high perceived utility (51).

The *Comfort Ability* is a psychoeducational and skill-based intervention for adolescents with chronic pain and their caregivers. The *Comfort Ability* teaches teens and families about chronic pain and cognitive behavioral skills for pain management and functional improvement. The workshop has demonstrated improvements in functioning, depressive symptoms, and pain catastrophizing that are maintained at 1-month follow-up. Prior to the COVID-19 pandemic, the *Comfort Ability* was delivered in a 6-h, 1-day workshop, with youth and parents meeting separately. During the pandemic, the workshop switched to virtual delivery via telehealth (length and number of sessions variable across locations; e.g., six 1-h sessions, four 2-h sessions), with some locations continuing to offer the virtual delivery modality in addition to the in-person workshop. The *Comfort Ability* is currently delivered in 23 children's hospitals internationally. Information on how to adopt the workshop can be found on the *Comfort Ability* website (39). The cost of the workshop is variable, with some locations charging out-of-pocket costs ranging from roughly $150 to $300 and others billing insurance.

*Empowered Relief* is a one-session pain relief skill intervention for adults with acute and chronic pain. The 2-h intervention is delivered by interprofessional certified instructors (see above, clinician trainings). The standardized intervention is delivered to groups either in-person or online via Zoom or another conference platform. *Empowered Relief* is didactic and includes pain neuroscience education. The participants acquire three core pain management skills and complete a personal plan for empowered relief. Participants also receive a binaural audio app for integration into their personal plans and for daily use. An NIH-funded randomized controlled trial (RCT; *N* = 263) revealed that single-session *Empowered Relief* was non-inferior to 16 h of cognitive behavioral therapy at 3 months post-treatment for reducing pain catastrophizing, pain intensity, pain interference, pain bothersomeness, depression, anxiety, fatigue, and sleep disturbance (52). A second RCT compared online received *Empowered Relief* to Usual Care in 105 patients with mixed etiology chronic pain (53). The results revealed high patient engagement and satisfaction, in addition to a similar pattern of reductions in pain intensity, pain interference, pain catastrophizing, pain bothersomeness, anxiety, and sleep disturbance at 3 months post-treatment.

To date, 800 certified instructors are delivering *Empowered Relief* in 43 US states, 25 countries, and seven languages (Canadian French, Spanish, Italian, Dutch, Danish, Italian, and English). Separate versions of *Empowered Relief* exist for chronic pain and acute/surgical pain, and certification flexibly allows clinicians to deliver either or both versions. *Empowered Relief* is being delivered as “standard care” at multiple healthcare organizations, including Cleveland Clinic Spine Surgery and the Neurological Institute (54), the Phoenix VA, Cedars-Sinai Health Care, Lehigh Valley Health Network, Allegheny Health Network, Brigham and Women's Hospital, the Canadian VA, the NHS in the UK, and Humana Neighborhood. Standard care means that all patients are recommended to receive Empowered Relief, rather than the traditional model of psychological intervention wherein patients are typically screened for treatment or referred. A standard and uniform approach allows institutions to emphasize the applicability of the information and intervention to all patients, thereby destigmatizing it and boosting patient engagement. Empowered Relief is the subject of five in-progress clinical trials being conducted by five different principal investigators with the research funded by the NIH or the Canadian Institutes for Health Research. A PCORI-funded national comparative effectiveness national trial is underway in which 1,200 adults with chronic pain of any type are being randomized to online one-session Empowered Relief vs. online eight-session CBT (75). The goal of this research is to test which online and home-based treatment works best and for whom and to determine the heterogeneity of treatment effects for key subpopulations across a diverse national patient population. The development of *Empowered Relief* for Youth is underway. Patients may access the intervention through their healthcare systems, in the community, and publicly through online national registration offered by some certified instructors.

Another promising intervention is the one-session Mindfulness-Oriented Recovery Enhancement (MORE) for people with chronic pain and opioid use disorder, developed by Handley and Lingard (59). MORE includes aspects of mindfulness training, third-wave CBT, and principles from positive psychology into an integrative intervention approach. Coping strategies focus on mindfulness training to target automatic habit behavior and foster nonreactivity, positive reappraisal training to regulate negative emotions and nurture a sense of meaning in life, and training in savoring pleasant events and emotions to ameliorate deficits in positive affectivity. The one-session MORE resulted in reductions in pain up to 3 months post-intervention. In addition, a one-session Pain Psychology and Neuroscience Self-Evaluation Internet Intervention (PPN) was developed by Kohns et al. (60). PPN focuses on personalized pain neuroscience education where patients are engaged in exercises to evaluate various psychosocial risk factors with respect to their pain. PPN resulted in reductions in pain intensity and interference at 1-month post-intervention, but the results were not maintained at 10 months.

Finally, the one-session Emotional Awareness and Expression Training (EAET) (pain, stress, and emotions “PSE” class) was developed by Ziadni et al. for adults with chronic pain (57). EAET involves 2 months of therapist-delivered weekly 2-h-long treatment sessions (eight sessions; 16 h total). EAET advocates a pain treatment model in which pain can be substantially reduced by helping people resolve emotional problems that amplify or generate pain (76). Unresolved childhood trauma, relationship problems, and psychological conflicts augment the “danger alarm” of bodily pain via the brain's predictive coding. EAET includes emotional disclosure, emotional awareness/expression exercises, and relationship communication changes, all of which are thought to reduce fear and pain. EAET is a newer therapy, and several trials provide evidence of pain reduction and improved function (77). PSE is a distilled version of a longer-course EAET and is a 2-h one-session intervention that is manualized and delivered by doctoral-level psychologists with EAET training. The standardized intervention is delivered to patient groups either in person or via Zoom or another conference platform at the individual's home. PSE comprises didactic content (i.e., pain psychology and neuroscience education) and an interactive and experiential component designed to help patients practice emotional regulation skills. Participants also complete a personalized prescription plan with their individual goals.

Altogether, these brief clinician-delivered interventions can provide rapid access to care, particularly when delivered online, with early evidence for several interventions suggesting strong effects at 3 months post-treatment. Owing to their low-burden and often home-based formats, one-session behavioral treatments are likely to appeal to patients, providers, and insurers and enhance treatment engagement and completion. These interventions could help shift patient understanding of their pain and enhance readiness for pain self-management. Single-session treatments also be integrated and routinely offered in primary, specialty pain care settings and even possibly as a prerequisite to costly and invasive procedures (e.g., surgery).

### 3.5. Efficiency trends for historically intensive in-patient pain treatment

In-person interdisciplinary team (IDT) care has been cited as the gold-standard treatment approach because it tends to address each component of the established biopsychosocial model (78); however, it is typically offered inpatient and is resource-intensive and burdensome. An innovative practice at the Salem VA Healthcare System, primarily serving rural Appalachian Veterans, offers Veterans a lower burden brief interdisciplinary team care via shared appointments with the Veteran, support person, and a five-discipline interdisciplinary team, as well as monthly telephonic support. The model, called PREVAIL Center for Chronic Pain Interdisciplinary Track, also integrates whole healthcare, which was recently highlighted by the National Academies of Sciences, Engineering, and Medicine (79). While PREVAIL is not as brief and scalable as other innovations described in this article, it nicely illustrates the overall trend of leveraging technology and applying novel clinical efficiencies to meet the needs of a complex population. The PREVAIL Interdisciplinary Track is a 6-month program that involves standardized patient education, an initial in-person meeting with the Veteran, support person, and an interdisciplinary team (IDT; psychology, interventional pain, physical therapy, nutrition, and pharmacy) that develops a patient-centered, whole health, biopsychosocial treatment plan, monthly phone calls with a whole health coach, and a 6-month follow-up meeting with the Veteran, support person, and the IDT. This program was launched in January 2022, and, to date, more than 200 Veterans have completed the initial evaluation. Given the emphasis on shared decision-making within the IDT initial evaluation and tailoring the treatment plan to the patient, no treatment plan has ever been duplicated. The program's use of technology, namely, telehealth visits for the patient education component and phone coaching, also reduces patient time burden which may enhance access for Veterans that experience barriers to traditional care. This model also lowers the healthcare system burden by offering an interdisciplinary approach that avoids the resources associated with inpatient treatment (e.g., staffing, space) and requires fewer scheduling calls compared to traditional models that have patients meeting with providers individually. While this approach is still being studied, early findings support patient acceptability, and high satisfaction rates have been demonstrated: 9.2/10 (*N* = 176 Veterans and caregivers who completed the initial IDT evaluation in 2022). With the PREVAIL reducing the burden for both patients and the healthcare system, it is hoped that the program may be scaled across the VHA and expand Veteran access to biopsychosocial pain care.

### 3.6. Digital, on-demand treatments

Fully automated behavioral treatments offer the benefit of rapid scaling. Here, we review several on-demand pain treatments and the supporting evidence for each.

#### 3.6.1. Virtual reality

In-clinic and in-patient virtual reality (VR) has long shown analgesic effects for procedural-related pain (80–82). In recent years, VR has been adapted to treat other pain conditions, including fibromyalgia and chronic low back pain (64–68). An early study compared VR for chronic pain to the same therapeutic content delivered in an audio-only format (no VR or visual display) (66). The study findings revealed that while patient engagement in both modalities was similar, the VR group evidenced superior analgesic benefits post-treatment, thus suggesting that the VR modality potentially offers unique benefits. This early study led to the creation of the 8-week VR treatment device described below.

An FDA-authorized prescriptive VR device offers a home-based sequential multimodal self-administered immersive (3D) treatment (64). The 56-day involves daily VR sessions lasting 6–8 min each, for a total of ∼50 min per week for 8 weeks. The program incorporates evidence-based self-regulatory skills used in cognitive behavioral therapy for chronic pain (diaphragmatic breathing, biofeedback elements, cognition, and emotion regulation), mindfulness principles, and pain education. Researchers conducted an RCT that included 188 community adults with chronic low back pain to compare the VR therapeutic program to a VR sham that involved 2D non-skill content delivered through the same model of VR headset. The VR therapeutic program results revealed clinically meaningful reductions in pain intensity and multidimensional pain interference with effects superior to VR sham. Moreover, clinical benefits were sustained at 3, 6, and 24 months post-treatment, with nominal regression to the mean at distal follow-up timepoints (64, 67, 68). The benefits of therapeutic VR include the devices being mailed directly to patients’ homes for on-demand self-administered treatment. Thus, prescriptive VR overcomes many of the primary barriers to access seen for traditionally delivered treatments and allows clinicians a convenient way to prescribe evidence-based behavioral pain care. In 2023, the Centers for Medicare and Medicaid Services (CMS) created the first HCPCS Level II health procedure billing code for a virtual reality program for chronic low back pain, describing the treatment device as durable medical equipment and creating a pathway for Medicare and commercial payer coverage.

#### 3.6.2. Empowered relief for surgery

Earlier in this article content discussed the live instructor delivered of 1-session Empowered Relief (either in-person or via Zoom). To extend to surgical populations, *Empowered Relief* was tailored to the surgical context and digitized into video-based modules that patients could receive on-demand at home, in the clinic, or in the hospital after surgery. An RCT was conducted on women undergoing breast cancer surgery to compare the digital intervention (then called *My Surgical Success*) to a health education control intervention that involved no active pain relief skills (55). Women who engaged with *My Surgical Success* were found to require about 1 week less of opioids after breast cancer surgery relative to women in the control group, suggesting benefits for reducing the time to opioid cessation after surgery.

Researchers next conducted an RCT of the digital intervention in 84 orthopedic trauma surgery patients, with the majority receiving their assigned treatment on an iPad in the hospital on post-operative days 1–3 (56). Patients who received the digital 45-min version of Empowered Relief reported significantly less pain after surgery relative to controls, and the analgesic benefits persisted for 3 months after surgery. The results suggested that engagement with Empowered Relief produced clinically meaningful and sustained analgesia and enhanced recovery after surgery. Moreover, the results underscored the potential for low cost, low burden, brief education, and pain self-regulatory skills to alter the trajectory of surgical recovery.

### 3.7. Self-guided Internet and app-based interventions

WebMAP Mobile (49) is a self-guided app-based intervention developed from an 8-week Internet-based intervention, focused on teaching youth skills for chronic pain management relaxation training, cognitive strategies, sleep, and activity engagement. WedMAP Mobile reduced pain intensity and functional impairment and is freely available for Apple and Android. iCanCope is another web-based educational intervention for youth with pain (83). iCanCope seeks to empower youth by providing information related to chronic pain, as well as various evidence-based treatment modalities (e.g., physical therapy, pain psychology), and provides behavioral modification skills for various lifestyle domains (e.g., sleep, physical activity). An app-based version of iCanCope is currently the focus of the ongoing study. Current work is also underway on the development of a digital graded exposure treatment (GET) for youth with chronic pain (iGET Living) (44), targeting pain-related impairment by supporting youth in engaging in previously avoided activities. Adapted from an interdisciplinary outpatient GET (GET Living) (84), iGET Living aims to provide a self-paced intervention that youth could engage with daily (∼10 min/day) over the course of 6 weeks, during which they learn about chronic pain, the rationale for value-based activity exposures, and practice engaging in activities they are avoiding due to fear of pain. Current work is focused on the finalization of a prototype of iGET Living that is expected to undergo an examination of feasibility and preliminary effectiveness in the coming year. During the feasibility trial, the value of therapist involvement (as opposed to self-guided) will be systematically evaluated to inform the finalization of a scalable intervention that can be feasibly implemented into healthcare.

*PainTrainer* is an open-access website app that teaches evidence-based pain coping skills using a self-administered, home-based software program (62). The system delivers eight weekly sessions via any online platform. The digital curriculum covers progressive muscle relaxation, pacing, pleasant activity scheduling, recognizing negative automatic thoughts, pleasant imagery and distraction, problem-solving, and maintenance strategies. *PainTrainer* was studied in a participant-blinded trial of patients with chronic pain. At post-treatment, greater increases in function, pain coping, and global improvement were found for *PainTrainer* compared to a control condition. Benefits persisted at 52 weeks, and 91% of participants (older adults, largely from rural, low-income areas) completed all eight sessions (85). Rini et al. (64) found similar results in a controlled trial in patients with painful arthritis that demonstrated improved self-efficacy, reduced anxiety, and less pain-related interference with functioning.

Magee and colleagues in Australia have developed brief video intervention and text-based support for patients undergoing prescription opioid tapering (69). The intervention was co-designed with patients and aims to enhance patient self-efficacy for opioid tapering and tapering outcomes.

Finally, with 40%–90% of patients with advanced cancer experiencing pain, improved access to behavioral pain treatment is needed. As one important step, Dr. Desiree Azizoddin and US colleagues have developed gamified CBT for the palliative context (86) and a CBT-based mobile health intervention (app) for patients with cancer (71). The initial results of the CBT app suggest favorable patient appraisal, and two efficacy trials are currently underway (87, 88). Such smartphone-delivered interventions hold promise for delivering scalable patient education, pain management skills, daily text messaging, and other key supports for patients with cancer pain.

## 4. Discussion

A variety of innovations are needed to address the diverse needs of people who have pain. This article, while not exhaustive, reviewed several innovations that are offering patients and clinicians new avenues for training, treatment, and resources. New directions that can expand the portfolio of accessible pain care include brief and effective behavioral pain treatments that leverage technology via telehealth and fully automated interventions. There are four key areas of future research that could grow the impact of these innovations: (1) expanding the pain workforce, (2) improving dissemination and implementation, (3) using precision medicine to understand treatment selection, and (4) exploring necessary patient-centered tailoring.

There is a need to train a wide range of healthcare specialties in delivering pain education and behavioral pain care, and technological solutions can offer flexible and accessible opportunities to deliver widespread education. Successful education models have utilized telemedicine to provide pain education, case-based learning, and consultative services to clinicians treating adults and children with pain (89). Clinician decision support tools embedded into medical record systems can provide strategies for when and how to make appropriate pain treatment referrals and patient-centered prescription opioid stewardship (90). Asynchronous training modules and online workshops also offer opportunities to disseminate pain education in graduate and resident training programs and diverse medical settings (91). Building from these educational efforts, patients and clinicians would benefit from further research examining how to expand the dissemination and implementation of these educational tools across *all* healthcare settings and to *all* healthcare providers who may treat patients with pain. Examination of which specific formats and tools are most effective in each medical setting is needed. Further, the development and implementation of strategies to increase clinician engagement, translate knowledge into practice, and sustain long-term improvements are needed to ensure pain education and training are most effective. Strategies might include incentivizing clinician participation, standardizing pain education in medical training, and providing regular training opportunities available through chronic pain workgroups and consultation services (92).

In addition, the integration of pain education for patients and families across healthcare settings is needed. For example, approximately 60%–70% of patients that present to the emergency department have pain, and most report low pain management satisfaction (93). Those utilizing emergency services for pain are more likely to have worse chronic pain and psychological wellbeing (94). Few hospital settings offer pain education or behavioral pain care prior to or following surgical procedures, even though there is evidence that brief behavioral interventions can lower healthcare expenditures and improve surgical outcomes (95). Primary barriers include poor insurance coverage, high out-of-pocket costs, and the lack of flexible, patient-centered treatments that can scale within these settings (96). Several treatments detailed, including *Empowered Relief* (52, 56) and WebMap (49), may be viable pain treatments that could be freely available and easily integrated into a variety of medical settings. Additional research into the development, tailoring, and implementation of digital behavioral pain management tools is needed to continue expanding access and effectiveness across settings.

Effective patient-centered pain treatments must also be flexible and responsive to patient needs. Technology-enhanced digital pain treatments are designed to be delivered flexibly and can overcome access barriers. However, improving precision pain medicine with digital treatments, such as when, to whom, and at what dose of treatment, is needed. For example, depression, anxiety, and insomnia are highly comorbid in chronic pain and are associated with worse pain treatment outcomes (97). Pain catastrophizing is also a robust predictor of poor pain outcomes (98). Some patients may likely benefit from integrated pain treatments that can also improve comorbid depression, anxiety or insomnia, or targeted CBT skills that focus on reducing pain catastrophizing. Yet, little is known about who may respond most effectively to which treatment length or modality, and exploration of patient phenotypes and their impact on treatment responsiveness would greatly improve our ability to deliver the right treatment to the right patient. Additionally, *when* treatment is delivered is likely to be an important factor. Early exposure to biopsychosocial pain care is associated with a reduced risk of acute to chronic pain transition, and those with worse disability and psychological comorbidities are at the highest risk for developing chronic pain (99). Therefore, identifying patients at the highest risk of developing chronic pain and providing pain treatments before invasive procedures may improve patient outcomes and reduce healthcare expenditures.

Lastly, treatments must be patient-centered and ensure that patients feel welcomed, understood, and respected. To do this, the use of patient-centered, first-person language is crucial to help patients understand the biopsychosocial nature of chronic pain without feeling as though they are being blamed or exaggerating their pain. Additionally, the integration of cultural adaptations can help patients from different backgrounds feel seen and heard. For example, Indian and Chinese populations describe cultural responses to pain, such as suppressing pain responses, to be important to address along with the inclusion of spiritual and holistic approaches beyond traditional Western treatments (100). People who experience race-based trauma and stress (RBTS) are also disproportionately at higher risk for developing chronic pain. Developing and tailoring treatments that are sensitive to the needs of specific racialized groups and address cultural, structural, and institutional factors that result in RBTS and pain (101). Intersecting factors, such as stigma and medical mistrust, toward behavioral pain treatments and clinicians are also important to address to increase treatment engagement. Digital treatments offer a unique opportunity to reduce biases and misinterpretation of pain experiences, which contribute to poor pain outcomes (102). Additionally, digital interventions that are low-burden, accessible, and skill-based may mitigate stigma toward the use of behavioral interventions in chronic pain care.

Innovative digital pain education and treatments are capable of transforming how evidenced-based pain care is delivered to clinicians and patients and are uniquely situated to reduce many access, dissemination, and implementation barriers that many face. Further research into how these technological advancements can be implemented in a wide variety of medical settings and effectively serve diverse patient populations will enhance patient outcomes and reduce the societal burden of pain.
